# Bis(2-hy­droxy­ethanaminium) naphthalene-1,5-disulfonate

**DOI:** 10.1107/S1600536811023269

**Published:** 2011-06-30

**Authors:** Cong Wang, Sheng Li Yang

**Affiliations:** a College of Pharmaceutical Sciences, Zhejiang University of Technology, Hangzhou 310014, People’s Republic of China

## Abstract

In the crystal structure of the title compound, 2C_2_H_8_NO^+^·C_10_H_6_O_6_S_2_^2−^, the anion lies on an inversion centre. The components are held together by O—H⋯O hydrogen bond, forming a 2:1 aggregate. The aggregates are further connected by N—H⋯O and C—H⋯O hydrogen bonds.

## Related literature

For related structures, see: Gao *et al.* (2005 ▶); Li & Chai (2007 ▶); Russell *et al.* (1997 ▶); Sakwa & Wheeler (2003 ▶); Wang *et al.* (2008 ▶); Zhang *et al.* (2005 ▶).
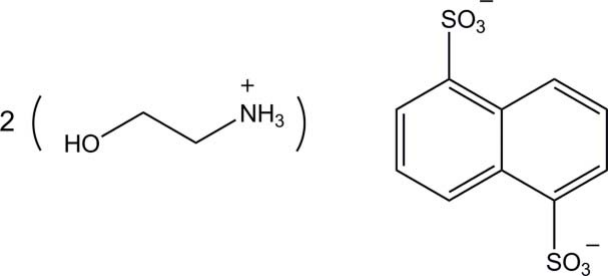


## Experimental

### 

#### Crystal data

2C_2_H_8_NO^+^·C_10_H_6_O_6_S_2_^2−^
*M* *_r_* = 410.48
Monoclinic, 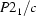

*a* = 9.7946 (14) Å
*b* = 8.9011 (13) Å
*c* = 10.4050 (16) Å
β = 104.334 (2)°
*V* = 878.9 (2) Å^3^
*Z* = 2
Mo *K*α radiation
μ = 0.35 mm^−1^
*T* = 293 K
0.42 × 0.34 × 0.30 mm

#### Data collection

Bruker APEX area-detector diffractometer
Absorption correction: multi-scan (*SADABS*; Bruker, 2001 ▶) *T*_min_ = 0.855, *T*_max_ = 0.898
4721 measured reflections
1718 independent reflections
1625 reflections with *I* > 2σ(*I*)
*R*_int_ = 0.019

#### Refinement

*R*[*F* ^2^ > 2σ(*F* ^2^)] = 0.029
*wR*(*F* ^2^) = 0.080
*S* = 1.04
1718 reflections
122 parameters
1 restraint
H atoms treated by a mixture of independent and constrained refinement
Δρ_max_ = 0.43 e Å^−3^
Δρ_min_ = −0.34 e Å^−3^

### 

Data collection: *SMART* (Bruker, 2002 ▶); cell refinement: *SAINT* (Bruker, 2002 ▶); data reduction: *SAINT*; program(s) used to solve structure: *SHELXS97* (Sheldrick, 2008 ▶); program(s) used to refine structure: *SHELXL97* (Sheldrick, 2008 ▶); molecular graphics: *SHELXTL* (Sheldrick, 2008 ▶); software used to prepare material for publication: *SHELXL97*.

## Supplementary Material

Crystal structure: contains datablock(s) global, I. DOI: 10.1107/S1600536811023269/is2725sup1.cif

Structure factors: contains datablock(s) I. DOI: 10.1107/S1600536811023269/is2725Isup2.hkl

Supplementary material file. DOI: 10.1107/S1600536811023269/is2725Isup3.cml

Additional supplementary materials:  crystallographic information; 3D view; checkCIF report

## Figures and Tables

**Table 1:** Hydrogen-bond geometry (Å, °)

*D*—H⋯*A*	*D*—H	H⋯*A*	*D*⋯*A*	*D*—H⋯*A*
O4—H4′⋯O2	0.835 (10)	2.041 (10)	2.840 (2)	160 (2)
N1—H1*A*⋯O4^i^	0.89	2.13	2.934 (2)	149
N1—H1*B*⋯O1^ii^	0.89	1.90	2.766 (2)	164
N1—H1*C*⋯O3^iii^	0.89	1.96	2.837 (2)	168
C3—H3⋯O1^iv^	0.93	2.41	3.270 (2)	154
C5—H5⋯O2^v^	0.93	2.55	3.459 (2)	167

Symmetry codes: (i) 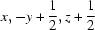; (ii) 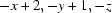; (iii) 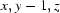; (iv) 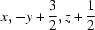; (v) 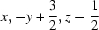.

**Fig. 1.**
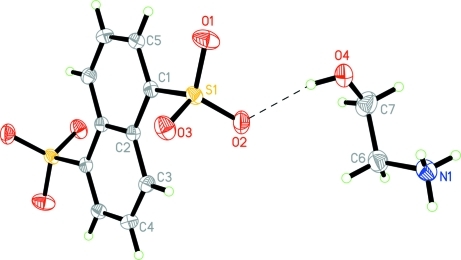
The molecular structure of the title compound. The unlabeled half of naphthalene-1,5-disulfonate anion are generated from the labeled half by symmetry operation of (1 - x, 2 - y, - z). The O—H···O hydrogen bond is illustrated as a dashed line.

**Fig. 2.**
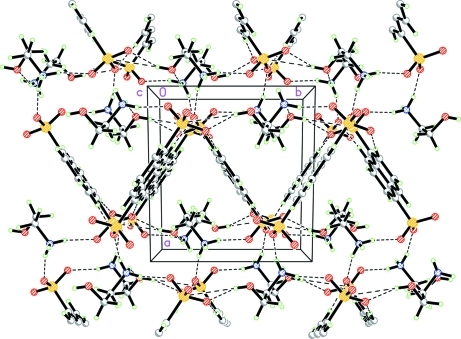
The crystal packing of the title compound, viewed down the c axis. Hydrogen bonds are drawn as dashed lines.

## supplementary crystallographic information

### Comment

Several supramolecular structures of naphthalene-1,5-disulfonate have been reported previously (Russell *et al.*, 1997; Zhang *et al.*, 2005; Gao *et al.*, 2005; Wang *et al.*, 2008). As an extension of research, we report here the synthesis and structure of the title compound, (I) (Table 1 & Fig. 1).

The naphthalene-1,5-disulfonate anion is linked to the ethanolaminium cation by O2—H4'···O4 hydrogen bond (Fig. 1 & Table 1). The crystal structure is further built by N—H···O and C—H···O hydrogen bonds. (Fig. 2 & Table 1). Due to steric hindrance of sulfonate, the nearest centroid separation between naphthalene rings is of 5.264 (3) Å, suggesting no π–π stacking. The arrangement of naphthalene rings is very similar to those observed in previous cases (Sakwa & Wheeler, 2003; Li & Chai, 2007; Wang *et al.*, 2008), but is different from that in bis(oxonium monohydrate) naphthalene-1,5-disulfonate, which shows a partial π–π stacking.

### Experimental

Naphthalene-1,5-disulfonic acid (5.1 g) and ethanolamine (1.2 g) in a molar ratio of 1:1 were mixed and dissolved in sufficient ethanol by heating to 373 K, at which point a clear solution resulted. The system was then cooled slowly to room temperature. Crystals of (I) (4.7 g) were formed, collected and washed with ethanol.

### Refinement

All H atoms except atom H4' were placed in calculated positions and allowed to ride on their parent atoms with distances of 0.93 Å for aromatic group, 0.97 Å for methylene and 0.89 Å for amino group, and with *U*_iso_(H) set at 1.2 or 1.5*U*_eq_ of the parent atoms. The positions of atom H4' were refined with a distance restraint O—H = 0.84 (2) Å, and with *U*_iso_(H) = 1.5*U*_eq_(O).

### Crystal data

**Table:**

2C_2_H_8_NO^+^·C_10_H_6_O_6_S_2_^2^^−^	*F*(000) = 432.0
*M**_r_* = 410.48	*D*_x_ = 1.551 Mg m^−^^3^
Monoclinic, *P*2_1_/*c*	Mo *K*α radiation, λ = 0.71073 Å
Hall symbol: -P 2ybc	Cell parameters from 1567 reflections
*a* = 9.7946 (14) Å	θ = 2.1–16.1°
*b* = 8.9011 (13) Å	µ = 0.35 mm^−^^1^
*c* = 10.4050 (16) Å	*T* = 293 K
β = 104.334 (2)°	Prism, colorless
*V* = 878.9 (2) Å^3^	0.42 × 0.34 × 0.30 mm
*Z* = 2	

### Data collection

**Table:**

Bruker APEX area-detector diffractometer	1718 independent reflections
Radiation source: fine-focus sealed tube	1625 reflections with *I* > 2σ(*I*)
graphite	*R*_int_ = 0.019
φ and ω scans	θ_max_ = 26.0°, θ_min_ = 2.2°
Absorption correction: multi-scan (*SADABS*; Bruker, 2001)	*h* = −12→11
*T*_min_ = 0.855, *T*_max_ = 0.898	*k* = −7→10
4721 measured reflections	*l* = −11→12

### Refinement

**Table:**

Refinement on *F*^2^	Secondary atom site location: difference Fourier map
Least-squares matrix: full	Hydrogen site location: inferred from neighbouring sites
*R*[*F*^2^ > 2σ(*F*^2^)] = 0.029	H-atom parameters constrained
*wR*(*F*^2^) = 0.080	*w* = 1/[σ^2^(*F*_o_^2^) + (0.0441*P*)^2^ + 0.3961*P*] where *P* = (*F*_o_^2^ + 2*F*_c_^2^)/3
*S* = 1.04	(Δ/σ)_max_ = 0.001
1718 reflections	Δρ_max_ = 0.43 e Å^−^^3^
122 parameters	Δρ_min_ = −0.34 e Å^−^^3^
1 restraint	Extinction correction: *SHELXL97* (Sheldrick, 2008), Fc^*^=kFc[1+0.001xFc^2^λ^3^/sin(2θ)]^-1/4^
Primary atom site location: structure-invariant direct methods	Extinction coefficient: 0.017 (3)

### Special details

**Table:**

Geometry. All e.s.d.'s (except the e.s.d. in the dihedral angle between two l.s. planes) are estimated using the full covariance matrix. The cell e.s.d.'s are taken into account individually in the estimation of e.s.d.'s in distances, angles and torsion angles; correlations between e.s.d.'s in cell parameters are only used when they are defined by crystal symmetry. An approximate (isotropic) treatment of cell e.s.d.'s is used for estimating e.s.d.'s involving l.s. planes.
Refinement. Refinement of *F*^2^ against ALL reflections. The weighted *R*-factor *wR* and goodness of fit *S* are based on *F*^2^, conventional *R*-factors *R* are based on *F*, with *F* set to zero for negative *F*^2^. The threshold expression of *F*^2^ > σ(*F*^2^) is used only for calculating *R*-factors(gt) *etc*. and is not relevant to the choice of reflections for refinement. *R*-factors based on *F*^2^ are statistically about twice as large as those based on *F*, and *R*- factors based on ALL data will be even larger.

### Fractional atomic coordinates and isotropic or equivalent isotropic displacement parameters (Å^2^)

**Table:**

	*x*	*y*	*z*	*U*_iso_*/*U*_eq_	
O1	0.84543 (12)	0.76241 (16)	−0.12460 (11)	0.0408 (3)	
O2	0.75476 (13)	0.65670 (12)	0.04999 (12)	0.0380 (3)	
O3	0.89350 (12)	0.88364 (13)	0.09087 (11)	0.0368 (3)	
O4	0.85710 (15)	0.38353 (14)	−0.03184 (13)	0.0449 (3)	
H4'	0.843 (3)	0.4733 (14)	−0.015 (2)	0.067*	
S1	0.79615 (4)	0.79324 (4)	−0.00698 (3)	0.02542 (15)	
N1	0.91425 (15)	0.17298 (16)	0.20858 (14)	0.0359 (3)	
H1A	0.9313	0.1641	0.2963	0.054*	
H1B	0.9898	0.2108	0.1872	0.054*	
H1C	0.8947	0.0830	0.1713	0.054*	
C1	0.64066 (14)	0.90372 (15)	−0.06386 (14)	0.0232 (3)	
C2	0.56077 (14)	0.95467 (15)	0.02605 (13)	0.0223 (3)	
C3	0.59815 (15)	0.91921 (17)	0.16367 (14)	0.0266 (3)	
H3	0.6775	0.8609	0.1983	0.032*	
C4	0.51843 (16)	0.97004 (17)	0.24527 (14)	0.0292 (3)	
H4	0.5440	0.9455	0.3349	0.035*	
C5	0.60178 (16)	0.94103 (17)	−0.19575 (14)	0.0271 (3)	
H5	0.6551	0.9074	−0.2526	0.033*	
C6	0.79262 (19)	0.2745 (2)	0.16027 (18)	0.0394 (4)	
H6A	0.8137	0.3716	0.2029	0.047*	
H6B	0.7112	0.2338	0.1856	0.047*	
C7	0.7569 (2)	0.2955 (2)	0.01220 (19)	0.0490 (5)	
H7A	0.7502	0.1977	−0.0300	0.059*	
H7B	0.6653	0.3433	−0.0157	0.059*	

### Atomic displacement parameters (Å^2^)

**Table:**

	*U*^11^	*U*^22^	*U*^33^	*U*^12^	*U*^13^	*U*^23^
O1	0.0307 (6)	0.0605 (8)	0.0332 (6)	0.0100 (5)	0.0116 (5)	−0.0071 (6)
O2	0.0458 (7)	0.0233 (6)	0.0464 (7)	0.0058 (5)	0.0145 (5)	0.0026 (5)
O3	0.0315 (6)	0.0343 (6)	0.0396 (6)	0.0016 (5)	−0.0006 (5)	−0.0078 (5)
O4	0.0649 (8)	0.0265 (6)	0.0475 (7)	0.0058 (6)	0.0219 (6)	0.0020 (5)
S1	0.0249 (2)	0.0249 (2)	0.0263 (2)	0.00367 (13)	0.00597 (15)	−0.00329 (13)
N1	0.0386 (7)	0.0330 (7)	0.0368 (7)	−0.0016 (6)	0.0102 (6)	−0.0055 (6)
C1	0.0238 (7)	0.0200 (7)	0.0262 (7)	−0.0003 (5)	0.0070 (5)	−0.0013 (5)
C2	0.0246 (7)	0.0194 (6)	0.0233 (7)	−0.0017 (5)	0.0063 (5)	−0.0007 (5)
C3	0.0274 (7)	0.0253 (7)	0.0261 (7)	0.0033 (6)	0.0050 (6)	0.0025 (6)
C4	0.0361 (8)	0.0305 (8)	0.0210 (7)	0.0014 (6)	0.0068 (6)	0.0026 (6)
C5	0.0325 (8)	0.0252 (7)	0.0256 (7)	0.0004 (6)	0.0109 (6)	−0.0017 (6)
C6	0.0420 (9)	0.0340 (9)	0.0454 (9)	0.0024 (7)	0.0168 (8)	−0.0010 (7)
C7	0.0594 (12)	0.0359 (10)	0.0457 (10)	−0.0083 (8)	0.0016 (9)	0.0037 (8)

### Geometric parameters (Å, °)

**Table:**

O1—S1	1.4485 (11)	C2—C2^i^	1.429 (3)
O2—S1	1.4533 (12)	C3—C4	1.365 (2)
O3—S1	1.4535 (11)	C3—H3	0.9300
O4—C7	1.417 (2)	C4—C5^i^	1.406 (2)
O4—H4'	0.835 (10)	C4—H4	0.9300
S1—C1	1.7862 (14)	C5—C4^i^	1.406 (2)
N1—C6	1.481 (2)	C5—H5	0.9300
N1—H1A	0.8900	C6—C7	1.505 (3)
N1—H1B	0.8900	C6—H6A	0.9700
N1—H1C	0.8900	C6—H6B	0.9700
C1—C5	1.371 (2)	C7—H7A	0.9700
C1—C2	1.4338 (19)	C7—H7B	0.9700
C2—C3	1.423 (2)		
			
C7—O4—H4'	107.6 (18)	C4—C3—H3	119.7
O1—S1—O2	111.79 (8)	C2—C3—H3	119.7
O1—S1—O3	113.50 (7)	C3—C4—C5^i^	120.96 (13)
O2—S1—O3	112.22 (7)	C3—C4—H4	119.5
O1—S1—C1	105.04 (7)	C5^i^—C4—H4	119.5
O2—S1—C1	107.12 (7)	C1—C5—C4^i^	120.20 (13)
O3—S1—C1	106.56 (7)	C1—C5—H5	119.9
C6—N1—H1A	109.5	C4^i^—C5—H5	119.9
C6—N1—H1B	109.5	N1—C6—C7	112.78 (15)
H1A—N1—H1B	109.5	N1—C6—H6A	109.0
C6—N1—H1C	109.5	C7—C6—H6A	109.0
H1A—N1—H1C	109.5	N1—C6—H6B	109.0
H1B—N1—H1C	109.5	C7—C6—H6B	109.0
C5—C1—C2	121.01 (13)	H6A—C6—H6B	107.8
C5—C1—S1	117.89 (11)	O4—C7—C6	113.31 (16)
C2—C1—S1	121.08 (10)	O4—C7—H7A	108.9
C3—C2—C2^i^	119.32 (15)	C6—C7—H7A	108.9
C3—C2—C1	122.72 (13)	O4—C7—H7B	108.9
C2^i^—C2—C1	117.97 (15)	C6—C7—H7B	108.9
C4—C3—C2	120.54 (13)	H7A—C7—H7B	107.7

Symmetry codes: (i) −*x*+1, −*y*+2, −*z*.

### Hydrogen-bond geometry (Å, °)

**Table:**

*D*—H···*A*	*D*—H	H···*A*	*D*···*A*	*D*—H···*A*
O4—H4'···O2	0.84 (1)	2.04 (1)	2.840 (2)	160 (2)
N1—H1A···O4^ii^	0.89	2.13	2.934 (2)	149
N1—H1B···O1^iii^	0.89	1.90	2.766 (2)	164
N1—H1C···O3^iv^	0.89	1.96	2.837 (2)	168
C3—H3···O1^v^	0.93	2.41	3.270 (2)	154
C5—H5···O2^vi^	0.93	2.55	3.459 (2)	167

Symmetry codes: (ii) *x*, −*y*+1/2, *z*+1/2; (iii) −*x*+2, −*y*+1, −*z*; (iv) *x*, *y*−1, *z*; (v) *x*, −*y*+3/2, *z*+1/2; (vi) *x*, −*y*+3/2, *z*−1/2.
